# Reassessing Plasmonic Interlayers: The Detrimental Role of Au Nanofilms in P3HT:PCBM Organic Solar Cells

**DOI:** 10.3390/polym17243262

**Published:** 2025-12-08

**Authors:** Alaa Y. Mahmoud

**Affiliations:** Department of Physical Sciences, College of Science, University of Jeddah, Jeddah 21589-80200, Saudi Arabia; aymahmoud8@uj.edu.sa

**Keywords:** organic solar cells, anodic layer, Au thin film, annealing, localized surface plasmon resonance, negative impact

## Abstract

This study examines the impact of incorporating a thin gold (Au) nanofilm as an interfacial buffer layer between the anode and the active layer in poly(3-hexylthiophene-2,5-diyl):[6,6]-phenyl-C_61_-butyric acid methyl ester (P3HT:PCBM) organic solar cells. A nominal 6 nm Au layer was thermally evaporated onto indium tin oxide (ITO) substrates and subsequently annealed at 550 °C for 30 and 60 min before completing the device fabrication. The optical, morphological, and electrical consequences of introducing these annealed Au films were systematically evaluated. Optical measurements revealed a marked enhancement in light absorption: the unannealed Au/P3HT:PCBM film showed a 54% increase at 560 nm, rising to 79% after 60 min of annealing, attributed to localized surface plasmon resonance. In contrast, electrical characterization indicated a decline in overall photovoltaic performance, with all parameters decreasing except for a modest 2% increase in fill factor. Atomic force microscopy further revealed that the actual Au nanofilm thickness was approximately 16 nm—significantly higher than the nominal 6 nm—leading to increased roughness and aggregation. The excessive thickness and roughened morphology of the annealed Au film likely hindered charge transport and reduced exciton generation by scattering and reflecting incident light away from the active layer. These findings highlight the competing effects of Au nanofilms: while they enhance optical absorption, they simultaneously degrade electrical performance. This underscores the importance of carefully optimizing nanofilm thickness and morphology to achieve a balanced interplay between plasmonic enhancement and electronic transport in organic solar cells.

## 1. Introduction

Organic solar cells (OSCs) have emerged as strong contenders to replace conventional silicon photovoltaics due to their distinctive advantages, including low-temperature and low-cost solution processing, lightweight nature, mechanical flexibility, and portability [1,2,3]. Among the established donor–acceptor material systems, poly(3-hexylthiophene-2,5-diyl) (P3HT) blended with the fullerene derivative [6,6]-phenyl-C61-butyric acid methyl ester (PCBM) remains one of the most extensively studied bulk-heterojunction (BHJ) combinations. The bicontinuous nanoscale network formed by P3HT (donor) and PCBM (acceptor) enables efficient exciton dissociation and charge transport, achieving single-junction efficiencies near 6% under standard illumination conditions (AM 1.5 G, 100 mW·cm^−2^, 25 °C) [4,5].

Considerable effort has been directed towards enhancing the power conversion efficiency (PCE) of OSCs through strategies such as developing new donor polymers and non-fullerene acceptors [6,7], and implementing tandem [8,9] or ternary architectures [10,11], leading to efficiencies approaching 10% [5]. However, OSCs still lag behind their inorganic counterparts, primarily due to the short exciton diffusion length of organic materials. Thin active layers are therefore required for efficient charge separation, but these simultaneously restrict light absorption [12,13,14,15].

To address this absorption–transport trade-off, the use of light-management nanostructures has received increasing attention. Plasmonic enhancement—arising specifically from noble metal nanostructures such as Au and Ag [16,17,18,19,20]—can amplify the local electromagnetic field through localized surface plasmon resonance (LSPR), thereby increasing photon harvesting within the active layer. In parallel, other non-plasmonic strategies, including quantum dots [21,22] and graphene-based additives [23,24] have also been employed to improve light absorption and charge transport through spectral conversion, scattering, and enhanced electrical conductivity.

Among plasmonic materials, gold nanoparticles and ultrathin Au films have shown notable capability in boosting light absorption due to LSPR, enabling electromagnetic field enhancement by orders of magnitude [25,26]. Our previous work demonstrated that incorporating gold nanorods into various device layers—including the active [2], buffer [1], and cathode [3] layers—can yield PCE improvements of up to 21%.

However, most studies [1,2,3,16,17,18,19,20,21,22,23,24,25] discussing metallic nanostructures report performance enhancement, while little attention has been given to cases where plasmonic layers may instead hinder efficiency. Variations in metal film thickness, surface roughness, and annealing-induced morphological evolution can introduce trap-assisted recombination, disrupt charge extraction pathways, or increase optical reflection, yet such drawbacks remain insufficiently explored in P3HT:PCBM systems. In particular, the interdependent effects of Au nanofilm thickness, nanoscale morphology, and annealing conditions on charge-collection dynamics at the anode interface have not been systematically examined.

Contrary to the common assumption that Au layers inevitably enhance absorption and PCE, our results show that a nominal 16 nm Au interlayer can reduce device efficiency despite increasing optical absorption. This work is therefore distinct in demonstrating that plasmonic Au nanofilms positioned directly beneath the active layer may have a detrimental impact on OSC performance due to exciton quenching, charge recombination, and interface-related losses. This perspective challenges the prevailing interpretation of plasmonic enhancement and emphasizes that enhanced light absorption does not necessarily translate into higher PCE without careful interfacial morphological control.

In this study, we systematically investigate the effect of incorporating a thin Au nanofilm into the anodic layer of P3HT:PCBM-based OSCs. A nominal 6 nm Au film was thermally evaporated onto indium tin oxide (ITO)-coated glass substrates and subsequently annealed at 550 °C for 30 and 60 min prior to the deposition of the remaining layers. The optical, morphological, and electrical characteristics of the fabricated devices were comprehensively analyzed to elucidate the dual role of Au nanofilms in modulating light absorption and charge transport within the active layer. While the Au nanofilm enhances the local electromagnetic field within the active layer, it simultaneously scatters/reflects part of the incoming light before it reaches the active layer. This underscores the need to optimize Au nanofilm morphology to balance optical benefits with electrical performance in organic solar cells.

## 2. Materials and Fabrication

### 2.1. Chemical Materials

All chemicals and materials were used as received without any further purification. The polymer P3HT with a high regioregularity of 98% was obtained from Rieke Metals. The fullerene derivative PCBM (>99.5%) and the solvent 1,2-dichlorobenzene (anhydrous, 99%) were supplied by Sigma-Aldrich. The hole transport and buffer layer material, poly(3,4-ethylenedioxythiophene):poly(styrenesulfonate) (PEDOT:PSS) (CLEVIOS™ P VP AI 4083), was purchased from HC Starck. Gold (Au 99.99%-metals basis), aluminum (Al, 99.99%-metals basis), and lithium fluoride (LiF 99.99%-metals basis) were obtained from Alfa Aesar.

**Figure 1:**
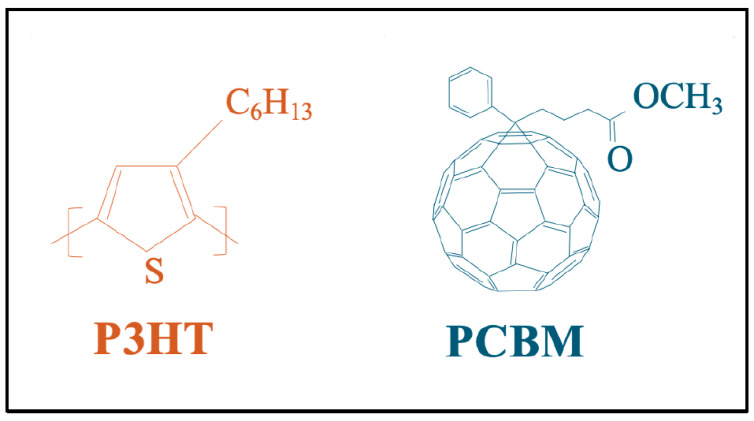
The chemical structures for the polymer P3HT and the fullerene PCBM.

Figure 1 illustrates the chemical structures of P3HT and PCBM, which together constitute the active layer of the fabricated organic solar cell devices.

### 2.2. Instrumentation

The surface structure was characterized using field emission scanning electron microscopy (FEG-SEM Hitachi S-4700, Hitachi High-Tech Corporation, Tokyo, Japan) with an accelerating voltage ~1–4 KV. The morphology was also tested using the scanning mode of atomic force microscopy (AFM NanoInk’s DPN 5000, NanoInk, Inc., Skokie, IL, USA). A double-beam spectrophotometer (PerkinElmer LAMBDA 650, PerkinElmer, Shelton, CT, USA) was used to measure the UV-vis spectra profiles. All tested samples were measured in the visible range of the spectra (~300–850 nm). A solar simulator (Oriel Instruments) of a xenon lamp and AM 1.5 G filter was used to characterize the organic solar cell devices under ambient conditions. A light meter (Bioscience LI-250, LI-COR, Inc., Lincoln, NE, USA) was used to calibrate the lamp’s intensity to 100 mW/cm^2^. An electrometer (Keithley 2400, Keithley Instruments, Solon, OH, USA) was used to measure the current-voltage output. A spin-coater (Laurell WS-650Mz-23NPPB, Laurell Technologies Corporation, Lansdale, PA, USA) was used to obtain the active layer film (P3HT:PCBM). A thermal evaporator (Tecuum VCM 600 V1, Tecuum AG, Winterthur, Switzerland) was used to evaporate Ag, Li and Al. A hotplate, ultrasonic bath and plasma etcher were also used in the fabrication process of OSCs.

## 3. Experimental Procedures

**Figure 2:**
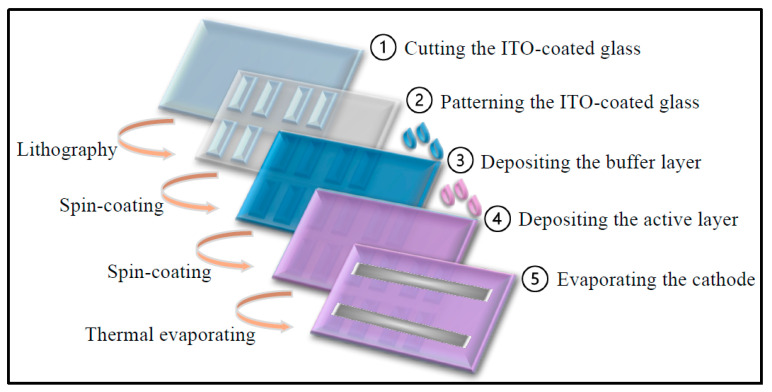
The fabrication steps of the OSC device.

The fabrication steps for the OSC devices are illustrated in Figure 2, following the procedures described in references [1,2,3]. ITO-coated glass substrates were first cut to the desired dimensions, patterned using a UV lamp, and thoroughly cleaned with a mild detergent solution. The substrates were then sequentially sonicated in acetone, isopropanol, and deionized water for 10 min each. After cleaning, they were dried under a nitrogen stream and baked at 150 °C for 20 min to remove residual moisture. Subsequently, the substrates underwent oxygen plasma treatment to enhance surface wettability and improve the adhesion of the following layers.

Next, the aqueous solution of the buffer layer material, PEDOT:PSS, was filtered through a 0.45 µm PVDF membrane and subsequently spin-coated onto the treated ITO substrates. The spin-coating process was carried out at 4000 rpm for 30 s, with an acceleration ramp of 10 s up to 1100 rpm. To ensure complete dehydration, the coated substrates were heated at 120 °C for 1 h and then transferred into a nitrogen-filled glove box for active-layer deposition. The P3HT:PCBM blend (20 mg/mL, 1:0.8 *w*/*w*) was prepared in 1,2-dichlorobenzene, filtered through a 0.45 µm PTFE membrane, and spin-coated onto the ITO/PEDOT:PSS stack. The deposition was performed in a three-step spin-coating sequence: 200 rpm for 5 s, 500 rpm for 5 s, and finally 1000 rpm for 60 s to form the BHJ active layer. The resulting wet films were placed in covered Petri dishes inside the nitrogen-filled glovebox and allowed to dry under ambient conditions for 30 min.

**Figure 3:**
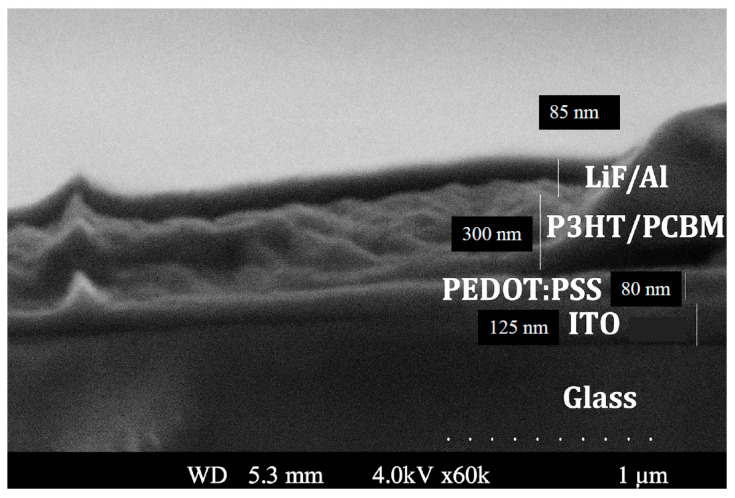
SEM micrograph of the pristine OSC device layers showing their thicknesses.

Finally, the cathode layer was deposited by thermal evaporation under high vacuum conditions (<10^−6^ Torr), forming a bilayer consisting of lithium fluoride (LiF, ~6 nm) followed by aluminum (Al, ~90 nm), with deposition rates maintained at ~1 Å/s for LiF and ~2.5–5 Å/s for Al. A shadow mask was used to define the electrode pattern. Each substrate hosted eight identical devices, with each device featuring an active area of 0.16 cm^2^. Figure 3 displays an SEM micrograph of the pristine OSC device layers showing their thicknesses.

**Figure 4:**
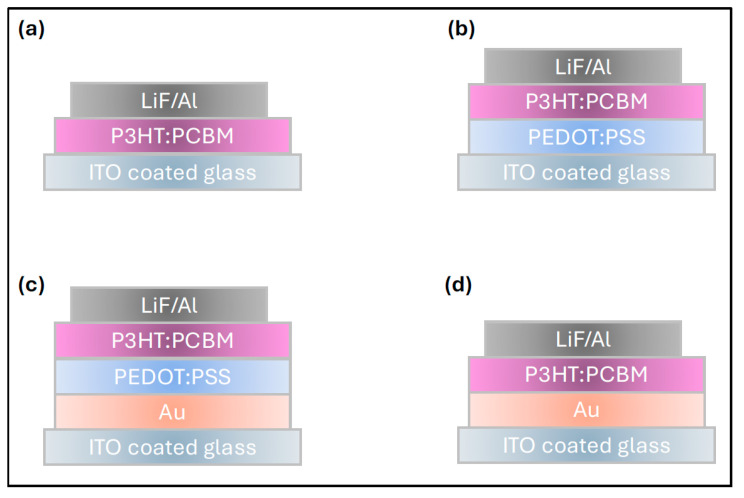
Schematic illustration of the four fabricated device configurations: (**a**) without both the Au nanofilm and the buffer layer, (**b**) with a buffer layer but without the Au nanofilm, (**c**) with both the Au nanofilm and the buffer layer, and (**d**) with the Au nanofilm but without the buffer layer.

For the devices incorporating the Au nanofilm, a gold layer with an approximate thickness of 6 nm was thermally evaporated onto the pre-cleaned ITO-coated glass substrates, with deposition rate maintained at ~2.5–5 Å/s. The coated substrates were then placed on a hotplate and annealed under an ambient condition at 550 °C for 30 and 60 min to induce nanostructural formation and improve film uniformity. Following the annealing process, the remaining device layers were sequentially deposited according to the fabrication procedure described above. The complete device architecture and layer configuration are presented in Figure 4.

## 4. Measurements and Discussion

### 4.1. The Optical Measurement of Au Nanofilms

**Figure 5:**
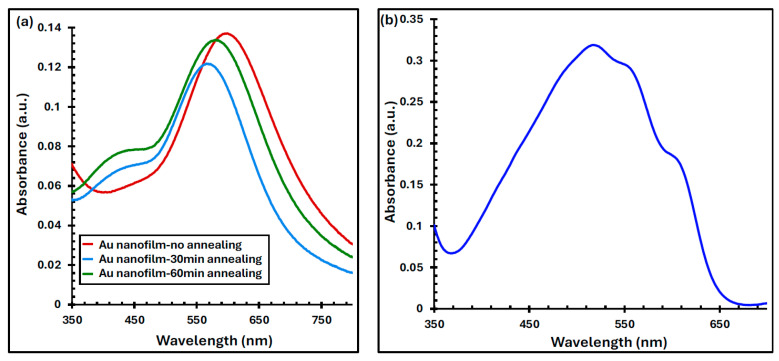
The absorbance profile for the (**a**) evaporated Au and (**b**) spin-coated P3HT:PCBM nanofilms.

**Figure 6:**
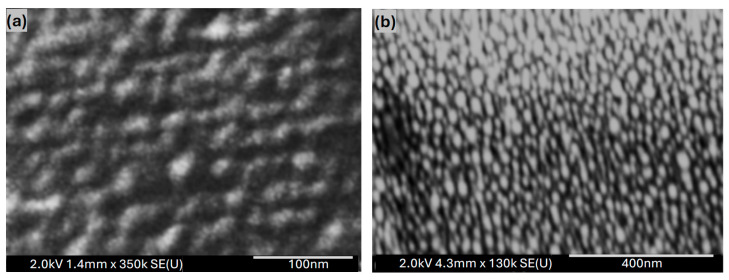
SEM images for Au nanofilm deposited on ITO-coated glass after annealing at 550 °C for (**a**) 30 and (**b**) 60 min.

**Figure 7:**
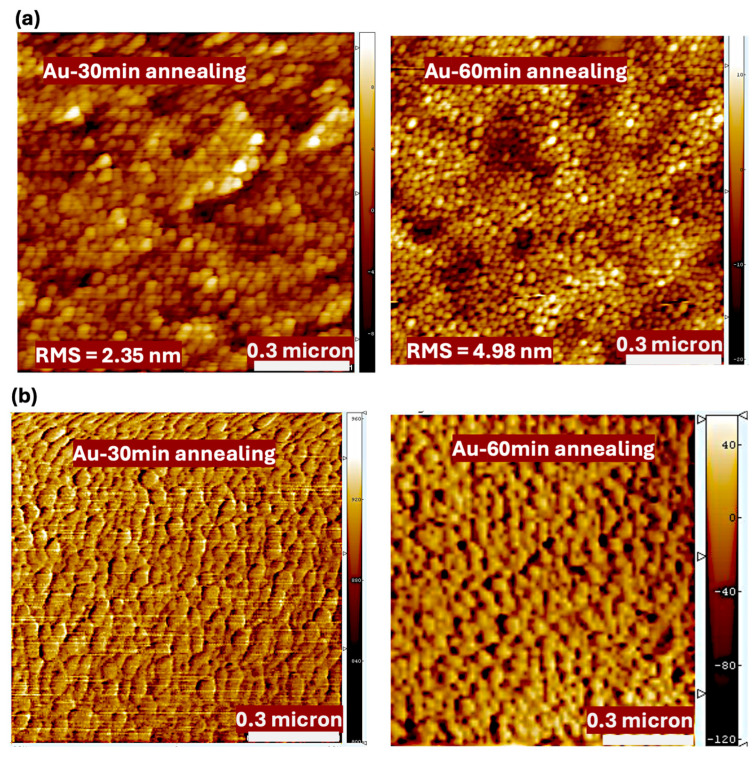
AFM (**a**) height and (**b**) phase images for the Au nanofilms annealed at 550 °C for 30 and 60 min.

Figure 5a presents the UV–Vis absorbance spectra of 6 nm-thick Au films before and after annealing at 550 °C for 30 and 60 min. The pristine Au nanofilm exhibits a distinct plasmonic absorption peak at 605 nm within the visible range. Upon annealing, the peak experiences a blue shift to 576 nm, accompanied by a reduction in absorption intensity of approximately 3% and 11% for 30- and 60-minute annealing durations, respectively. These plasmonic absorption peaks arise from LSPR—the collective oscillation of free electrons at the interface between two media of differing dielectric constants—which results in strong light absorption and scattering within the visible spectrum [27,28]. As confirmed by the SEM and AFM micrographs shown in Figure 6 and Figure 7, annealing the nanofilm promotes the transformation of the continuous Au layer into irregular or semi-spherical nanoparticles that sustain LSPR. The morphology, size, and surface density of these nanoparticles depend strongly on the annealing time and temperature [29,30,31].

The observed blue shift in the plasmonic peak after annealing can be attributed to the quantum confinement effect, indicating a reduction in the average nanoparticle size [32,33]. Moreover, the appearance of shoulder features in the absorption spectra of the annealed films (Figure 5a) suggests that the nanoparticles are not perfectly spherical, leading to the presence of a secondary resonance mode that is associated with multipolar plasmon excitation beyond the primary dipole mode [1,34]. When the annealing duration was further increased to 120 min, this secondary plasmonic peak became more pronounced, as shown in the Supplementary Figures S2–S4.

Importantly, the absorption band of the Au nanofilms overlaps significantly with that of the P3HT:PCBM active layer (400–650 nm), as illustrated in Figure 5b. This spectral overlap indicates the potential for plasmonic enhancement of light absorption within the active layer of the organic solar cells.

### 4.2. The Structural Measurement of Au Nanofilms

Figure 6 presents the SEM micrographs of the Au nanofilms annealed at 550 °C for 30 and 60 min, while Figure 7 shows the corresponding AFM images. Both SEM and AFM analyses reveal that annealing significantly alters the microstructure and surface morphology of the Au nanofilms, leading to the formation of discrete Au nanostructures.

As revealed from the AFM, prolonging the annealing duration from 30 to 60 min results in a pronounced increase in surface roughness, with the root-mean-square (RMS) roughness rising from 2.35 nm to 4.98 nm. This increase in roughness is attributed to the coalescence and growth of Au nanoparticles during thermal treatment, which induces morphological reconstruction and nanoparticle aggregation. The annealing time, therefore, serves as a key parameter in tuning the nanoparticle size and surface morphology, which in turn influences the crystallinity, optical absorption characteristics, and plasmonic behavior of the nanostructured Au films [35,36].

### 4.3. The Optical Properties of Au/P3HT:PCBM Films

**Figure 8:**
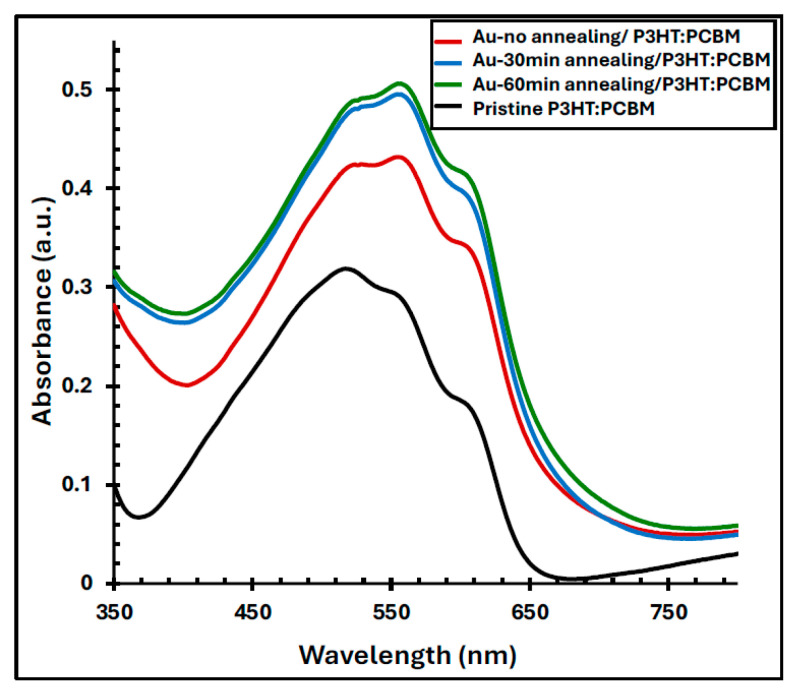
The absorbance profile for Au/P3HT:PCBM film before and after annealing at 550 °C for 30 and 60 min, along with the absorbance of the pristine P3HT:PCBM.

Figure 8 illustrates the UV–Vis absorption spectra of the pristine P3HT:PCBM film and the Au/P3HT:PCBM composite films, both before and after annealing at 550 °C for 30 and 60 min. The pristine P3HT:PCBM film exhibits a characteristic absorption band between 420 and 620 nm, with a primary absorption peak centered at 520 nm and two distinct shoulders at approximately 560 and 600 nm. These features correspond to the π–π* transitions and vibronic structures of the P3HT backbone, indicative of its degree of molecular ordering [4,37]. Upon incorporating the Au nanofilm (without annealing), the absorption spectrum shows a noticeable red shift and a significant enhancement in intensity, particularly at the 560 nm shoulder, which increases by approximately 54% and becomes the dominant peak. This enhancement arises from the plasmonic near-field effect of the Au nanostructures, which amplifies the local electromagnetic field within the active layer [27,38]. Further annealing of the Au/P3HT:PCBM films at 550 °C for 30 and 60 min results in an additional increase in absorption intensity, reaching up to 79% enhancement relative to the pristine P3HT:PCBM film. The improvement in optical absorption can be attributed to the optimized formation of Au nanoparticles during annealing, which promotes stronger LSPR coupling and enhanced light–matter interaction within the active layer.

### 4.4. The Effect of the Annealed Au Nanofilms on OSC Parameters

Since annealing the Au nanofilm for 60 min resulted in the highest enhancement of P3HT:PCBM film absorptivity, this condition was selected for integration into practical OSC devices. To investigate the influence of the Au nanofilm and PEDOT:PSS buffer layer on charge extraction and hole-collection efficiency at the anode interface, four sets of devices were fabricated simultaneously, for comparative analysis. These configurations, as previously illustrated in Figure 3, are: (a) **Without buffer layer**: ITO/P3HT:PCBM/LiF–Al (b) **With buffer layer**: ITO/PEDOT:PSS/P3HT:PCBM/LiF–Al, (c) **With Au and buffer layer**: ITO/Au/PEDOT:PSS/P3HT:PCBM/LiF–Al (d) **With Au but without buffer layer**: ITO/Au/P3HT:PCBM/LiF–Al.

**Figure 9:**
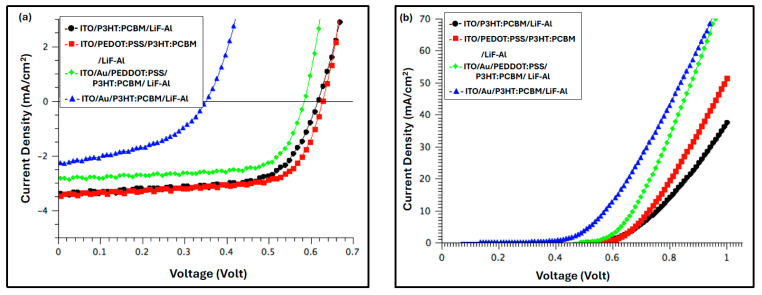
The IV characteristics for OSC devices with and without Au film, along with devices without Au or buffer films (**a**) under light and (**b**) in the dark.

Figure 9 presents the current density–voltage (J–V) characteristics of the devices under illumination (a) and in the dark (b), both before and after deposition of the Au film. The corresponding photovoltaic (PV) parameters—including open-circuit voltage (V_OC_), short-circuit current density (J_SC_), fill factor (FF), and power conversion efficiency (PCE)—are summarized in Table 1. The series resistance (R_S_) was extracted from the dark J–V curves at approximately 0.86 V.

The dark J–V characteristics of all fabricated devices (Figure 9b) demonstrate well-defined rectifying behavior, confirming good diode performance. Under illumination, the photovoltaic parameters summarized in Table 1 reveal that the best device performance was achieved for the configuration incorporating the PEDOT:PSS anodic buffer layer. This device exhibited a PCE of 1.6%, primarily attributed to a J_SC_ of 3.74 mA·cm^−2^. The recorded efficiencies were lower than standard values reported for P3HT:PCBM devices. This deficiency is attributed to inadvertent ambient exposure during glovebox-to-evaporator transfer and during electrical measurements, conditions under which thermal degradation of the active layer is known to occur [39,40,41].

Introducing the Au nanofilm beneath the buffer layer resulted in a modest enhancement of the FF, increasing from 67.67% to 69.09%. Notably, this value lies among the highest reported for P3HT:PCBM-based OSCs [12,42]. The improvement is attributed to the reduced R_S_, which promotes more efficient charge extraction and enhances charge transport at the anode interface. In contrast, minor reductions in both V_OC_ and J_SC_ were observed, which collectively led to a ~26% decrease in PCE relative to the reference device (device with PEDOT:PSS), despite the improved FF. In contrast, devices fabricated with the Au nanofilm but without the PEDOT:PSS buffer layer exhibited significantly degraded performance, characterized by reductions in all key photovoltaic parameters—V_OC_, J_SC_, FF, and PCE—indicating that the direct contact between the Au nanostructured layer and the active layer negatively affects charge extraction and device efficiency.

**Figure 10:**
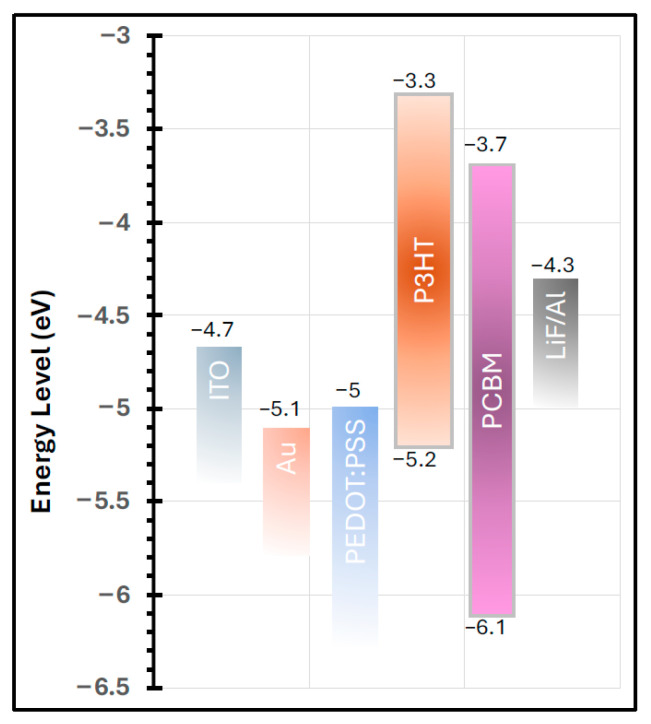
Schematic illustration of the energy band alignment of the materials used in the fabricated devices. The work function of the PEDOT:PSS buffer layer effectively facilitates hole extraction and transport toward the anode. A good alignment is observed between the work function of Au, the HOMO level of the P3HT polymer, and the ITO anode work function, which supports efficient charge transfer at the interface. The energy level values are adapted from reference [1].

Figure 10 presents the work functions of all materials incorporated in the fabricated devices, adapted from reference [1]. The work function of the PEDOT:PSS buffer layer is well aligned with both the work function of the ITO electrode and the highest occupied molecular orbital (HOMO) level of the P3HT polymer. This favorable energy-level alignment explains the superior performance of the device incorporating the buffer layer compared to the one without it. The effective matching of work functions facilitates efficient hole transport from the active layer to the anode, thereby enhancing charge collection and improving the overall device performance [43,44].

Although introducing the Au nanolayer between the ITO and the PEDOT:PSS buffer layer resulted in a 24% increase in J_SC_ and a 2% improvement in FF, the overall PCE decreased by 26%, primarily due to a 5% reduction in V_OC_. This decline in device efficiency was somewhat unexpected, considering that the annealed Au nanofilm significantly enhanced the optical absorption of the P3HT:PCBM active layer. The observed reduction in V_OC_ may be attributed to interfacial energy-level mismatches or increased recombination losses at the Au/buffer interface, which counteracted the optical gains provided by the plasmonic enhancement.

**Figure 11:**
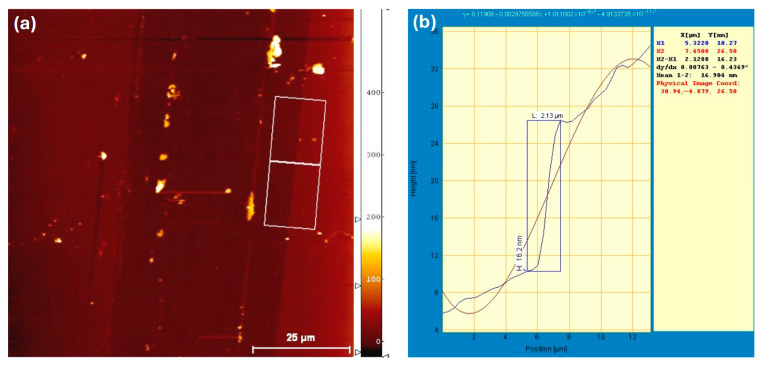
(**a**) AFM topographic image of the thermally evaporated Au nanofilm, and (**b**) corresponding height profile showing an average film thickness of approximately 16 nm.

This unexpected result prompted a closer examination of the actual Au film thickness. To verify the deposited thickness, AFM measurements were performed, as shown in Figure 11. The measured thickness was approximately 16 nm—significantly greater than the intended 6 nm. This discrepancy helps explain the performance degradation observed upon introducing the Au nanofilm. A relatively dense and continuous Au nanostructured layer at the anode interface can impede efficient hole collection and adversely affect optical absorption. Increased film thickness and surface coverage may obstruct photon penetration into the active layer, either through enhanced scattering or reflection of incident light away from the P3HT:PCBM region [45,46]. As a result, fewer excitons are generated, reducing the number of free charge carriers available for extraction and contributing to the observed decrease in V_OC_ and overall device efficiency.

In addition, the decline in device performance—despite the favorable work-function alignment of Au and the strong absorptivity of the Au/P3HT:PCBM system—can be attributed to the interplay between Au film conductivity and morphology. The electrical conductivity of thermally evaporated Au thin films is highly sensitive to post-deposition annealing conditions. Both annealing temperature and duration critically influence the film microstructure, affecting grain size, crystallinity, defect density, and film continuity, all of which govern electron transport. Typically, increasing annealing temperature or time promotes grain growth and reduces grain-boundary scattering, ultimately improving charge mobility and conductivity up to an optimal level. Beyond this optimum, however, ultrathin Au layers undergo pronounced morphological transformations—including agglomeration, island formation, void development, and interdiffusion at the substrate interface—which disrupt film continuity and substantially degrade electrical conductivity. The onset of such instabilities is strongly thickness-dependent and influenced by substrate interactions and surface energy. As a result, there exists a narrow processing window in which moderate annealing enhances electrical pathways, whereas excessive annealing induces morphological instability that suppresses electrical performance [47,48,49].

Furthermore, the morphological and charge-transport behavior of Au films is equally affected by annealing. Thermal treatment typically promotes grain coarsening and surface diffusion, which can improve crystallinity but also leads to the development of nanoscale surface irregularities and increased roughness. These changes impair charge extraction at the anode interface through multiple mechanisms: increased roughness extends the effective extraction pathway, enhances carrier scattering, and introduces interfacial trap states that serve as non-radiative recombination centers. Additionally, roughened Au surfaces can distort the local electric-field distribution, disrupting charge equilibration and altering the balance between hole extraction and recombination [29,30,31,50,51,52,53,54]. These combined effects provide a consistent explanation for the ≈5% reduction in V_OC_ observed in Au-integrated devices, even in the presence of enhanced optical absorption.

## 5. Conclusions

In this study, we investigated the effect of incorporating a Au nanofilm as an interfacial layer between the ITO anode and the P3HT:PCBM active layer in OSCs. Thin Au films (6 nm) were thermally evaporated onto ITO-coated glass substrates and subsequently annealed at 550 °C for 30 and 60 min prior to the deposition of the remaining device layers. The influence of the annealed Au films on the device performance was systematically examined through optical, morphological, and electrical analyses.

The Au nanofilms demonstrated excellent transparency and electrical conductivity, with a distinct optical absorption feature at 605 nm attributed to the LSPR of Au nanostructures. Upon annealing, this peak blue-shifted to 576 nm, accompanied by a reduction in intensity of 3% and 11% for 30- and 60-minute annealing durations, respectively. SEM and AFM analyses revealed that annealing promoted nanoparticle growth, increased surface roughness, and enhanced crystallinity, leading to structural evolution in the Au nanofilms.

Photovoltaic measurements of devices with and without Au and/or buffer layers showed that the device incorporating the conventional anodic buffer layer exhibited the highest performance, achieving a PCE of 1.6%. Although the Au nanofilm possesses a work function that aligns well with both the ITO electrode and the HOMO level of P3HT, replacing the buffer layer with the Au nanofilm only improved the FF by 2%, while the overall PCE decreased. This decline in performance was unexpected, given the 79% enhancement in optical absorption of the P3HT:PCBM film upon introducing the annealed Au nanofilm.

The observed reduction in device efficiency can be attributed to morphological and interfacial effects induced by annealing. Grain growth and surface irregularities increase surface roughness, leading to delayed charge dissipation, formation of surface-state defects, and enhanced carrier recombination. AFM analysis further revealed that the actual thickness of the deposited Au nanofilm was approximately 16 nm—substantially higher than the intended value—resulting in excessive optical scattering and reflection at the anode interface. These effects limit the amount of light reaching the active layer, reducing exciton generation and charge collection efficiency.

Overall, while the incorporation of Au nanofilms modifies the optical and structural properties beneficially, excessive film thickness and surface roughness negatively influence charge transport and extraction. Future optimization of Au nanofilm thickness and annealing conditions may yield a balanced enhancement in both optical absorption and device performance.

## Figures and Tables

**Table 1:** The OSCs’ PV parameters with and without Au nanofilm, along with devices without Au or buffer films.

Device Configuration	V_OC_ (V)	J_SC_ (mA/cm^2^)	FF %	R_S_ (Ω cm^2^)	PCE%	Δ % PCE
ITO/P3HT:PCBM/LiF-Al	0.62	−3.37	65.59	0.53	1.4	−12%
ITO/PEDOT:PSS/P3HT:PCBM/LiF-Al	0.62	−3.74	67.67	0.38	1.6	-
ITO/Au/PEDOT:PSS/P3HT:PCBM/LiF-Al	0.59	−2.84	69.09	0.25	1.2	−26%
ITO/Au/P3HT:PCBM/Li-Al	0.35	−2.27	45.55	0.31	0.4	−77%

## Data Availability

The original contributions presented in this study are included in the article/Supplementary Material. Further inquiries can be directed to the corresponding author.

## Supplementary Materials

The following supporting information can be downloaded at: https://www.mdpi.com/article/10.3390/polym17243262/s1, Figure S1: The absorbance of the PEDOT:PSS buffer layer was measured before and after annealing, as well as before and after Au deposition, with annealing durations of 30 and 55 min. Annealing led to an overall increase in the absorbance of PEDOT:PSS, indicating improved optical density. Following Au deposition, a distinct secondary absorption peak emerged at approximately 540 nm, consistent with the plasmonic response of the ultrathin Au layer; Figure S2: The absorbance of the 1–6 nm Au nanofilms (without annealing) shows that increasing thickness produces a second peak near the UV edge of the spectrum, along with higher overall absorbance. The primary peak becomes broader and exhibits a noticeable red shift as the film thickness increases; Figure S3: The absorbance of the 1–3 nm Au nanofilms was measured before annealing and after annealing at 330 °C for 1 and 2 h. Annealing induces a blue shift in the main absorption peak, while increasing the nanofilm thickness leads to the gradual emergence of a second peak in the UV region; Figure S4: The absorbance of the 4–6 nm Au nanofilms was recorded before annealing and after annealing at 330 °C for 1 and 2 h. Annealing leads to the appearance of a distinct second peak in the UV region.
