# Supplementary material for: Reassessing Plasmonic Interlayers: The Detrimental Role of Au Nanofilms in P3HT:PCBM Organic Solar Cells
Source: Polymers (Basel). 2025 Dec 8;17(24):3262. doi: 10.3390/polym17243262 (PMC12736476; doi:10.3390/polym17243262)
Supplement: Supplementary file 1 [file polymers-17-03262-s001.zip › polymers-4026408-supplementary.pdf]

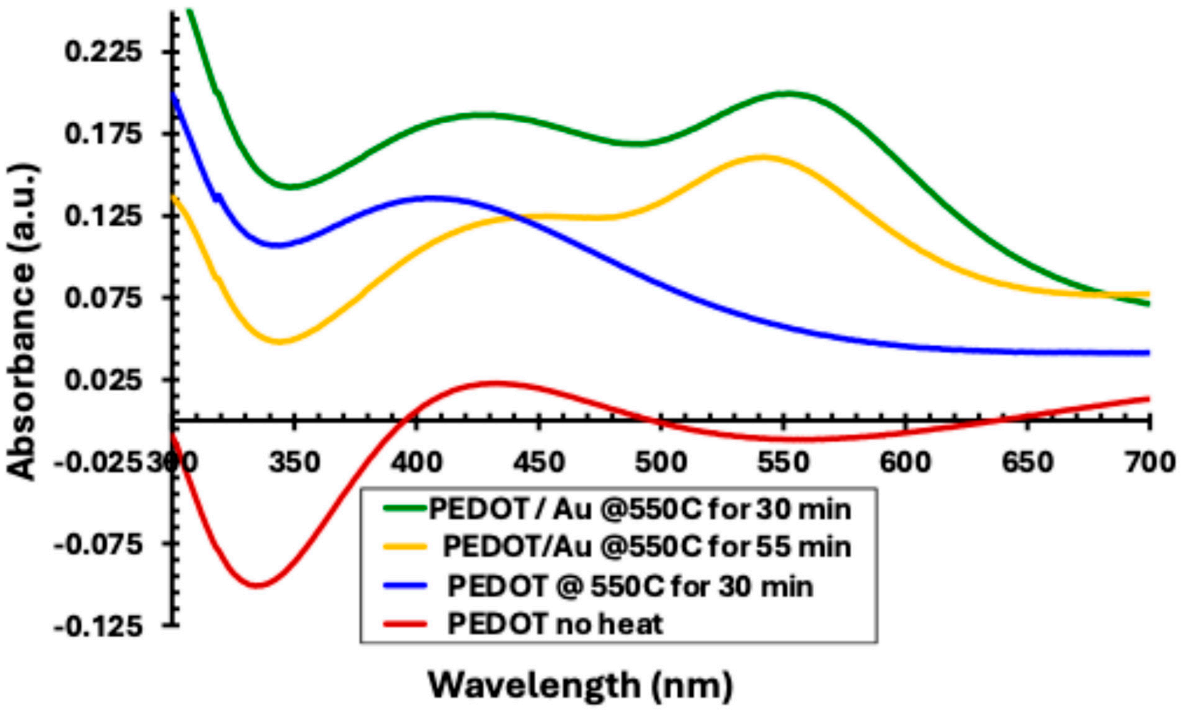


**Figure S1.** The absorbance of the PEDOT:PSS buffer layer was measured before and after annealing, as well as before and after Au deposition, with annealing durations of 30 and 55 minutes. Annealing led to an overall increase in the absorbance of PEDOT:PSS, indicating improved optical density. Following Au deposition, a distinct secondary absorption peak emerged at approximately 540 nm, consistent with the plasmonic response of the ultrathin Au layer.

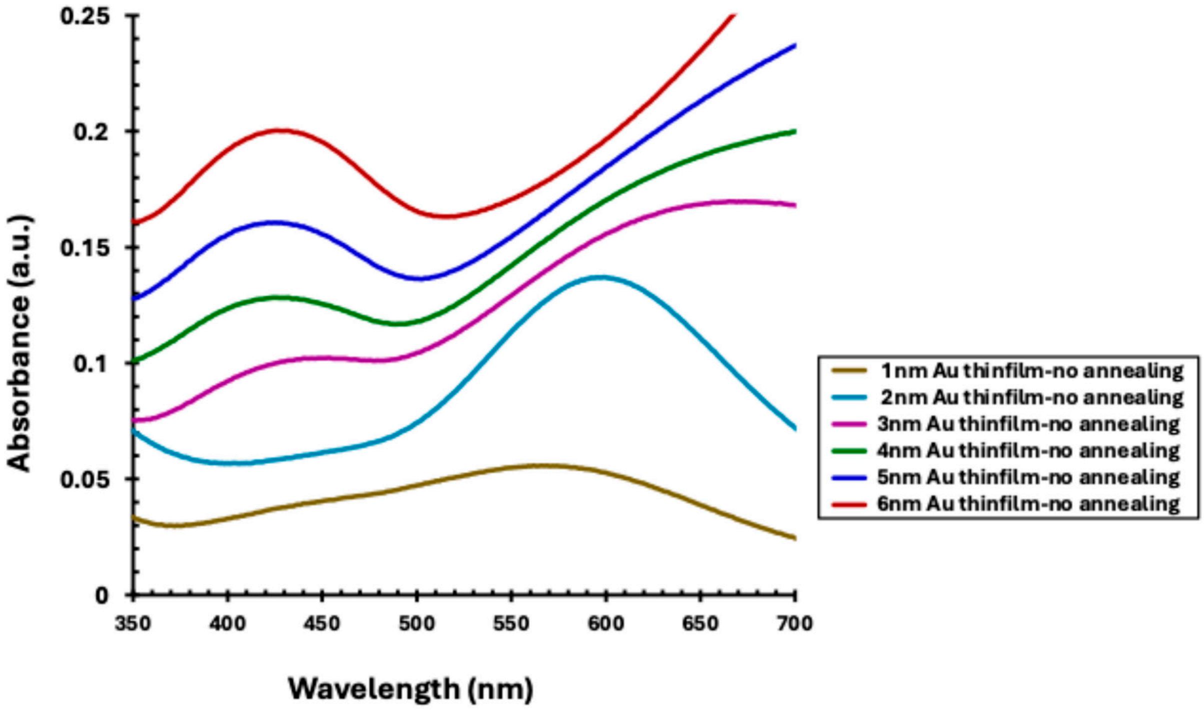


**Figure S2.** The absorbance of the 1–6 nm Au nanofilms (without annealing) shows that increasing thickness produces a second peak near the UV edge of the spectrum, along with higher overall absorbance. The primary peak becomes broader and exhibits a noticeable red shift as the film thickness increases.

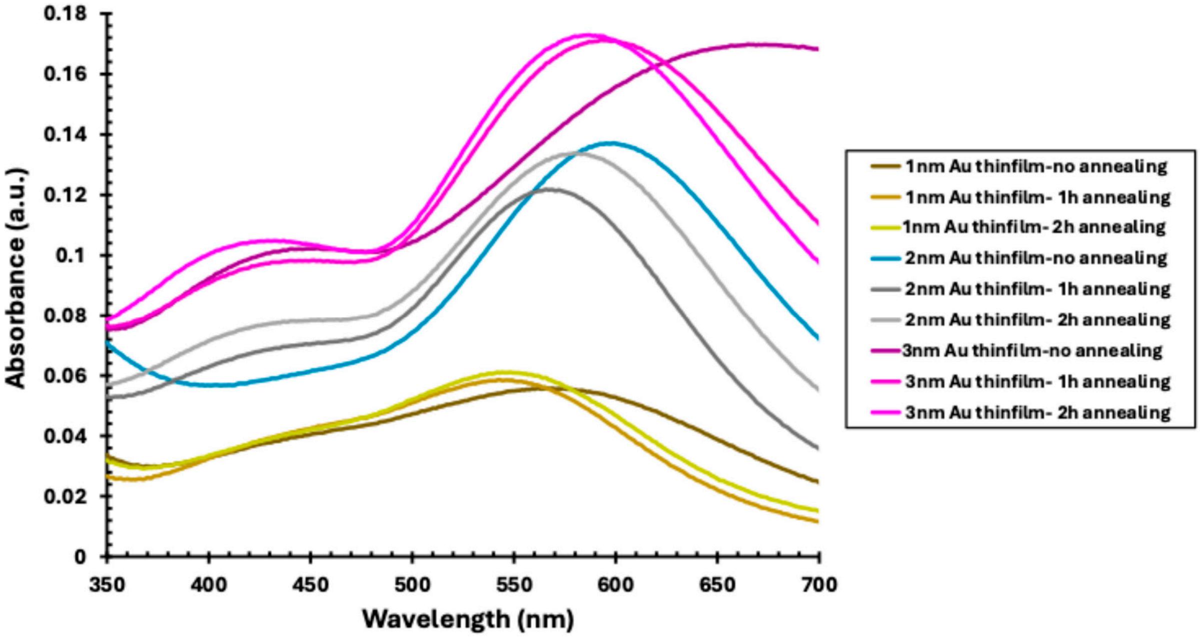


**Figure S3.** The absorbance of the 1–3 nm Au nanofilms was measured before annealing and after annealing at 330 °C for 1 and 2 hours. Annealing induces a blue shift in the main absorption peak, while increasing the nanofilm thickness leads to the gradual emergence of a second peak in the UV region.

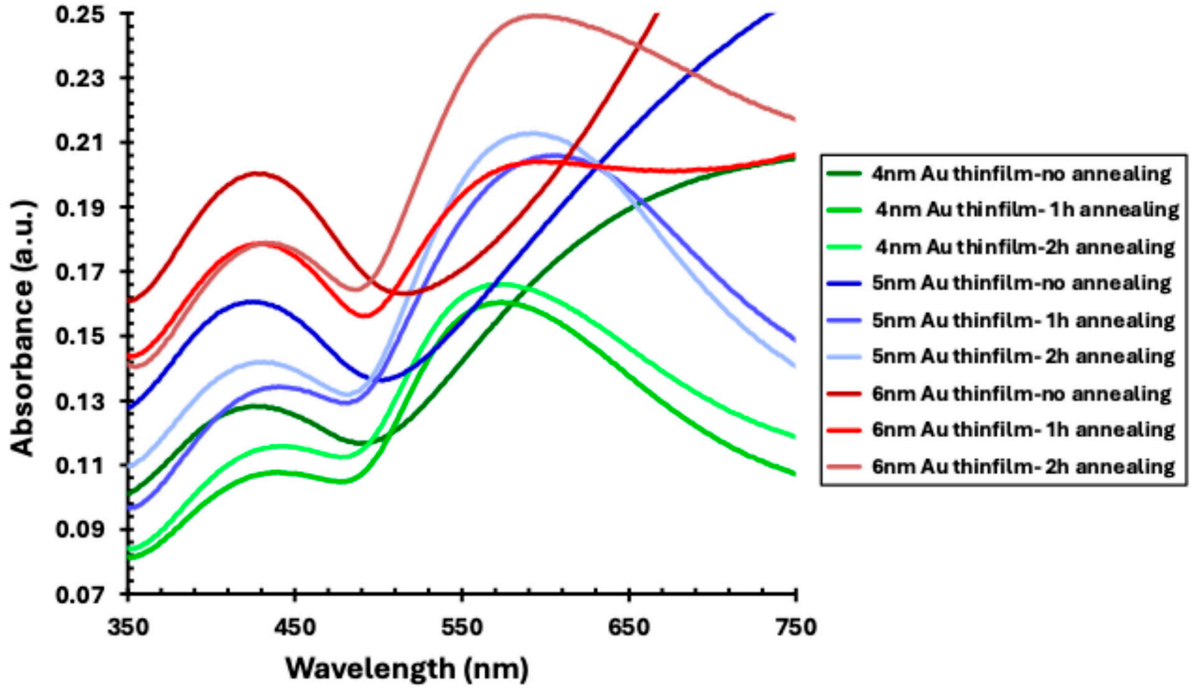


**Figure S4.** The absorbance of the 4–6 nm Au nanofilms was recorded before annealing and after annealing at 330 °C for 1 and 2 hours. Annealing leads to the appearance of a distinct second peak in the UV region.
